# A Review of Seven Hundred Cases of Acute Appendicitis
*Communicated to the Bath and Bristol Branch of the British Medical Association, 25th March, 1931.


**Published:** 1931

**Authors:** Hubert Chitty

**Affiliations:** Surgeon, Bristol Royal Infirmary, and Winford Hospital, Bristol


### The Bristol Medico-Chirurgical Journal

*"Scire est nescire, nisi id me*
*Scire alius sciret."*

AUTUMN, 1931.

### A REVIEW OF SEVEN HUNDRED CASES OF ACUTE APPENDICITIS.*

BY

HUBERT CHITTY, M.S., F.R.C.S.,

*Surgeon, Bristol Royal Infirmary, and Winford Hospital, Bristol.*

A VERY large proportion of the work of every general surgeon consists in the treatment of acute appendicitis, and these 700 cases represent those which have been under my care during the past eleven years at the Bristol Royal Infirmary and in private.

According to the reports of the Registrar-General, there are more than 3,000 deaths from this condition annually in this country alone, and the death-rate in the present series amounts to almost 5 per cent., so that it is well worth our while to consider why the mortality is so great and how it would be possible to reduce it.

Appendicitis is comparatively rare at the extremes of life. I have found an appendix abscess in a baby of 18 months, and I have seen one in the hernial sac

* Communicated to the Bath and Bristol Branch of the British Medical Association, 25th March, 1931.

of a man aged 91, though neither happens to come in the present series, among whom the youngest was aged $2\frac{1}{2}$ years and the oldest 86.

Among the 661 whose ages were noted the incidence is as follows :—

| | | | |
|---|---|---|---|
| First decade .. .. | 63 | Sixth decade .. .. | 37 |
| Second ,, .. .. | 202 | Seventh ,, .. .. | 18 |
| Third ,, .. .. | 168 | Eighth ,, .. .. | 9 |
| Fourth ,, .. .. | 93 | Ninth ,, .. .. | 5 |
| Fifth ,, .. .. | 66 | | |

*Causes of Death.*—There were thirty-four deaths, though not all of them are directly attributable to the disease with which the patient was admitted. One patient, for instance, was found post-mortem to be suffering from abdominal carcinoma, another died from diphtheria. Including the above, however, I find that twenty-four of the cases which died had been admitted with more or less extensive peritonitis, and that seventeen of these were so ill on admission that either they were not operated upon at all, or at most drainage was effected under local anæsthesia or with the aid of gas and oxygen. The other seven had an appendicectomy performed as well, and in the case of two of them an immediate drainage of the bowel was effected at the same time. In five of the remainder abscess formation had taken place. Of the remaining two, one was not operated upon. She was an old lady of 78, and her death was due to myocarditis. Mitral stenosis seems to have been the cause of the death of the last man, who died two hours after a perfectly straightforward and uncomplicated operation.

It would seem, therefore, that it should be possible almost to abolish deaths following appendicectomy if we could only get the cases early enough. In the present series there are only three deaths in cases operated upon within the first forty-eight hours after the onset of symptoms. In all of these the appendix was gangrenous and had perforated, and the fact that I drained the peritoneum of all three indicates that peritonitis was present. In one of them the fatal result was due in large measure to my failing to secure complete hæmostasis. Bleeding into an infected peritoneum is a most deadly complication, and I believe it accounts for at least one other death among the present series. There are undoubtedly others who died as the result of technical errors on my part, *e.g.* two diabetics who were operated upon without preliminary insulin treatment, and at least two patients in whom I removed the appendix when it was associated with abscess formation, and in so doing broke down protective adhesions and set up a general peritonitis. The fact remains, however, that if every case of acute appendicitis came into the hands of the surgeon during the first twenty-four hours deaths from the disease would become a negligible quantity.

An acute case submitted to operation on the first day should be up and about in ten days' time. Even a mild case takes that time to get well without operation, and still has an appendix which is almost sure to give rise to further trouble.

*Early Diagnosis.*—One of the most important contributions to the study of appendicitis which has appeared of late is a paper by Professor D. P. D. Wilkie, of Edinburgh,[1] who maintains that we have been including under the one head of appendicitis two totally different pathological conditions. The one is an inflammation of the mucous membrane and wall of the appendix which commonly subsides spontaneously in time, and at worst rarely does more than lead to local abscess formation. The other is an acute obstruction of the lumen of the appendix, generally by a fæcal concretion. It leads to a condition analogous to acute strangulation of a loop of gut, *i.e.* the lumen is distended with intensely poisonous decomposing intestinal contents, the wall rapidly becomes gangrenous and may perforate within twenty-four hours. No defensive adhesions have had time to form, and when the wall gives way the peritoneum is flooded with the most deadly infective material. Acute peritonitis rapidly follows, and it is this class of case which accounts for the majority of deaths. Every surgeon of experience must admit that all the above statements are perfectly correct.

Wilkie further maintains that the two conditions are clinically distinguishable. According to him, appendicitis proper begins with abdominal pain of gradually increasing severity, possibly some vomiting, a dirty tongue, and a steadily rising pulse and temperature. The obstructed appendix manifests itself by more intense pain, umbilical or epigastric in situation and colicky in nature. Vomiting is profuse. Pulse and temperature remain normal until perforation occurs. The symptoms, in fact, like the pathology, are those of acute intestinal obstruction.

For the past year or more I have been trying to distinguish these two conditions clinically, and I cannot do it. Some cases are true to type, others are not. The pain of an acutely inflamed appendix may be just as intense and its onset as sudden as that of an obstructed one, though the pain is apt to be more constant and less spasmodic. In some of my obstructed cases there has been remarkably little pain and little or no vomiting. Temperature and pulse are commonly quite low in the inflammatory cases during the first day—that precious twenty-four hours during which it is all-important that the diagnosis should be established. On the other hand, I have had gangrenous cases starting off with a rigor and a temperature of 103°, or with temperature and pulse rising steadily as in an inflammatory case.

One very important point emerges, however, viz. that it is quite common to have a gangrenous appendix on the point of perforating, yet for the patient to have a perfectly normal temperature and pulse. Pain and vomiting may have disappeared with the onset of gangrene. The patient, especially if a child, may have dropped off to sleep, so patient, friends, and doctor may well be in a fool's paradise till the appendix perforates and signs of peritonitis supervene. I saw one such case recently.

A small boy had been off colour for several days. On the previous day he had had some pain, though not severe, and had vomited once. During the day upon which I saw him he had had pain and tenderness in the right iliac fossa, and his pulse and temperature had been steadily rising, though the evening temperature was barely 100°. When I arrived the boy was asleep. Everything seemed to point to a mild inflammatory case. On principle I insisted upon bringing him in from the country and operating the same night. The appendix was gangrenous, oozing pus, and on the point of bursting. He had a stormy convalescence, and I doubt if he would have lived had he been left till the following day.

The general practitioner sends most of his appendix cases into hospital now as soon as he diagnoses them. As a consequence we see far fewer cases of general peritonitis than we did even a few years back. When we do see them they are generally due either to the patient not sending for a doctor early enough, or to failure of the doctor to diagnose the case as one of appendicitis until it has become one of peritonitis. I am quite certain that this failure is usually due to the fact that many practitioners are loth to diagnose appendicitis until a case presents all the signs and symptoms of the text-book description, particularly pain, tenderness, and rigidity in the right iliac fossa. Now the old text-books did not describe a case of appendicitis at all. The description applied to a case of localized peritonitis in the right iliac fossa. Rigidity in particular is never found until the peritoneum is affected. If we wish to lower our present death-rate we must endeavour to diagnose our cases before peritonitis has appeared.

In acute appendicitis no one sign or symptom is invariably present. Pain is almost always the first and is certainly the most constant symptom, though there is sometimes a history of malaise for a few days beforehand. The pain is usually referred to the umbilicus or epigastrium. Occasionally, especially in recurrent cases, it commences in the right iliac fossa. Exceptionally it may be referred to other regions, *e.g.* the loin, the groin, or even to the left side. Its degree of severity is very variable. I have seen it begin with the most intense colic, leading to collapse with a subnormal temperature. It is usually fairly bad, and yet not very uncommonly patients stroll up to the Casualty Department with a large appendix abscess from whom it is quite hard to elicit any history of pain whatever, and who may never have left their work. Absence of pain is most common in the very young, the very old, and in the mentally deficient.

Nausea is the next most common symptom, and vomiting occurs in three-quarters of the cases. Though constipation is the general rule, it is by no means constant, and it is well to remember that diarrhœa occurs in 5 per cent. of all cases. Diarrhœa is particularly common in cases where the appendix is situated in the pelvis, doubtless as the result of the local irritation it produces there.

No reliance can be placed upon pulse or temperature. The latter is rarely above 100° during the first twenty-four hours, and although one does occasionally find it higher than this, a temperature of 102° or more, even in a child, should make one very doubtful of the diagnosis.

It is quite exceptional to find a clean tongue, and I lay a good deal of stress upon the presence of a dirty one.

Pressure *over the appendix* almost always causes some pain, but this does not mean that one can elicit pain by pressing in the right iliac fossa. It is true that a good many appendices are to be found in this region, but a great many are tucked up behind the cæcum. Many more dip over the pelvic brim or are situated well within the pelvis, while exceptionally one may be found right up under the liver or even on the opposite side of the abdomen.

*Past History.*—It is remarkable how many cases which come to operation give a history of previous attacks of appendicitis. This is particularly the case in gangrenous appendicitis. Where the notes are reasonably full I can hardly find any such cases which failed to give either a definite history of previous attacks of appendicitis or of recurrent attacks of "indigestion," "biliousness," "gastric influenza," or abdominal pain which has masqueraded under some such label. It is extremely unlikely that a stricture and a fæcal concretion will be found in an appendix which has never been the seat of some inflammatory attack. This is a strong argument in favour of removing every appendix before it gets the chance of becoming inflamed a second time, however slight the first attack may be.

In every case of acute and persistent abdominal pain think first of appendicitis, and continue to regard this as the cause until you can definitely prove it to be something else. A dirty tongue and tenderness on deep pressure in any of the places where an appendix may be situated are the surest confirmatory signs in early cases. Do not wait for any others to appear if they do not happen to be already present.

*Pelvic Appendicitis.*—If the appendix were never to be found below the brim of the pelvis I am confident that considerably more than half of the deaths from appendicitis would be avoided. Apart from death, patients would be spared a vast amount of avoidable illness. Almost all the cases of general peritonitis admitted to hospital at the present time are of this ass. My notes show twelve of the thirty-four deaths as being due to pelvic appendicitis, and the number was undoubtedly greater, for the position of the appendix is only noted in a small proportion of cases. Time and again one gets the history that the doctor suspected appendicitis, but could find no signs in the abdomen. Of course not; there were no signs to find. So few realize that the appendix does not always inhabit the right iliac fossa, and that absence of abdominal signs in a case otherwise suggestive of acute appendicitis should indicate the imperative necessity for making a pelvic examination.

Apart from deaths and acute disasters due to gangrenous or suppurative pelvic appendicitis, the mild case which subsides in due course often infects the Fallopian tubes in the case of women, and is the origin of many cases of chronic backache and dysmenorrhœa. The gynæcologists remove almost (if not quite) as many chronically diseased appendices as the general surgeon does.

In this series I find nineteen cases of pelvic abscess. One burst spontaneously per vaginam and another per rectum. The rest I opened also per rectum. This is infinitely the best way of dealing with a localized pelvic collection and seems to be Nature's method also. It is much less dangerous than trying to get at it from above, and the drainage is more satisfactory. When the abscess approaches the rectum it causes rectal irritation, which manifests itself by the passage of numerous small stools containing mucus, and the abscess can then be evacuated easily and without danger.

*Treatment.*—The presence of an abscess rarely necessitates an immediate operation. When one is small and inaccessible it is better left alone. It will either come near the surface in time or it will burst into the bowel and cure itself. This is especially the case with deeply-placed residual abscesses. An operation which necessitates the separation of softened and adherent coils of gut may readily lead to a fæcal fistula, while it is difficult to effect satisfactory drainage.

An abscess rarely bursts into the general peritoneal cavity, though this did occur in one of the cases under review. She was admitted profoundly collapsed, with a temperature of 95° and a rigid and dull abdomen. A large abscess which had been wrapped round with omentum had suddenly flooded the abdomen with thick, yellow stinking pus. I removed the appendix and a good deal of omentum and swabbed up the pus, and the child made a perfectly uneventful recovery.

*Retrocœcal Abscess.*—The commonest site for an abscess is behind the cæcum and ascending colon.

The reason for this is that the retrocæcal appendix, being away from the peritoneal cavity, sets up no muscular rigidity when it is inflamed. Abdominal examination will only elicit pain when deep pressure is made over the ascending colon, and hence these cases are missed in the early stages. Missing a case of this kind does not matter so much as in the pelvic type. Such an one rarely sets up a general peritonitis. Still, once an abscess has formed several weeks are likely to be wasted in convalescence, and the chance of performing a clean appendicectomy has been lost.

*Errors in Diagnosis and Treatment.*—Of errors in diagnosis I will briefly mention only a few of the most common. Cases sent in to hospital labelled appendicitis are often found to be : (1) Renal infections and most commonly acute pyelitis; (2) acute cholecystitis; (3) early chest trouble, particularly basal pneumonia and diaphragmatic pleurisy ; (4) (much less commonly) infective gynæcological conditions such as pyosalpinx.

A case which really is one of acute appendicitis is rarely sent up labelled anything else, though I have myself diagnosed a ruptured appendix as a perforated duodenal ulcer and a case of high retrocolic obstructed appendix as one of renal colic.

Of errors in treatment much the most common is the giving of a purge. The general public has a great belief in castor oil as a remedy for every abdominal pain, and I regret to say that many doctors prescribe the same popular treatment. For a case of commencing appendicitis nothing could possibly be worse, and castor oil must be responsible for a number of deaths every year. If you must try to get the bowels open in a case of possible early appendicitis, a suppository or a simple enema is the only permissible method to employ.

SUMMARY.

If every case of acute appendicitis could be operated upon during the first twenty-four hours deaths from this disease could be almost completely abolished. At the present time most deaths are due to delay in diagnosis and treatment. The fault often lies with the patient who, when he has abdominal pain, sends for castor oil instead of for a doctor.

The commonest mistakes of the general practitioner are :—

(1) He knows that most cases subside if left alone, and so he waits till he finds that this particular case is not doing so. In consequence, he may sacrifice the patient's life or cause him many weeks of invalidity.

(2) He waits to make a diagnosis until signs of peritonitis have supervened, thus missing the most favourable time for operation.

(3) He does not know that pelvic appendicitis is common, produces no abdominal signs till very late, and can only be definitely diagnosed by rectal examination.

Of these three the last is nowadays the most frequent and fatal error. Gangrenous pelvic appendicitis accounts for most of the deaths, and it will continue to do so until a rectal examination becomes the invariable rule in every case of possible appendicitis in which physical signs are lacking in the abdomen.

In the most dangerous type of appendicitis (the obstructed variety) temperature and pulse frequently remain unaffected until perforation has taken place and all chance of performing a clean appendicectomy has gone. Early or late, once a case of appendicitis has been diagnosed it should be removed to a hospital or nursing home, and it should be left to the discretion of the surgeon whether an immediate operation is necessary or not.

My advice to a young surgeon would be: "Operate upon every case of acute appendicitis at once, with the exception of those which are seen late and already show signs of localized tumour formation. In those hold your hand until the attack has entirely cleared up or until an easily accessible abscess has formed. An early case with tumour formation requires operation, being due to omentum wrapped round an inflamed appendix. In draining an abscess do not try to remove the appendix if by so doing you run any risk of breaking down protective adhesions. Always make certain that you have secured absolute hæmostasis before you close your wound. When peritonitis makes drainage a necessity avoid putting tubes in amongst coils of intestine. It will usually suffice to put them down to or only just inside the peritoneum."

I have had many fewer cases of obstruction since adopting this method. When a patient is very ill with general peritonitis the less one disturbs the intestines the better. Drainage under a local anæsthetic or with the help of gas and oxygen and followed by the Fowler position, starvation, rectal subcutaneous or intravenous saline, and plenty of morphia gives better results than follow more extensive and radical operations. The mortality in such cases is bound to be high whatever the treatment adopted, and our one hope for better results than we are now getting lies in earlier diagnosis and earlier operation.

REFERENCE.

[1] *Brit. Med. Jour.*, 1931, i. 253.

